# Bilateral Linear Porokeratosis Treated With Topical Lovastatin 2% Monotherapy

**DOI:** 10.7759/cureus.43657

**Published:** 2023-08-17

**Authors:** Darlene Diep, Ilana A Pyatetsky, Kenneth L Barrett, Kamilah S Kannan, Kevin Wright, William Baker

**Affiliations:** 1 Family Medicine, University of California San Francisco Fresno, Fresno, USA; 2 Family Medicine, Burrell College of Osteopathic Medicine, Las Cruces, USA; 3 Family Medicine, Case Western Reserve University, Cleveland, USA; 4 Family Medicine, University of Georgia Athens, Athens, USA; 5 Dermatology, US Navy, San Diego, USA

**Keywords:** monotherapy, topical, lovastatin, linear porokeratosis, cholesterol

## Abstract

Linear porokeratosis is a rare skin disorder that presents along dermatomal or Blashko lines. While the mechanism of linear porokeratosis formation is unknown, both disrupted cholesterol synthesis and mevalonate accumulation have been proposed as possible theories. There is a small chance of transforming into cutaneous malignancies, most commonly squamous cell carcinomas. The patient is a 61-year-old male with an unusual presentation of bilateral linear porokeratosis. His condition provided a unique opportunity to compare the efficacy of topical treatments in a single individual. A previous trial had successfully cleared the porokeratosis plaques with topical cholesterol 2%/lovastatin 2% on the patient’s right arm. After a 12-week trial of topical lovastatin 2% monotherapy on the left arm, our current study demonstrated a comparable reduction of porokeratosis lesions. In our PubMed search, there has been a single reported case of disseminated superficial actinic porokeratosis successfully treated with topical lovastatin 2% monotherapy, but there have not been any reported cases of linear porokeratosis treated with this therapy. While topical lovastatin monotherapy for porokeratosis subvariants requires further studies, this case demonstrates similar efficacy of treating linear porokeratosis with topical lovastatin compared to cholesterol/lovastatin dual therapy. These findings support the theory of mevalonate accumulation as a more likely cause of linear porokeratosis compared to disruption of cholesterol synthesis.

## Introduction

Linear porokeratosis is a rare subtype of porokeratosis that is characterized by atypical hypertrophic keratinized plaques. The more common variants of porokeratosis include porokeratosis of Mibelli, disseminated superficial porokeratosis, porokeratosis palmaris et plantaris disseminata, punctate porokeratosis, and disseminated superficial actinic porokeratosis (DSAP) [1]. Of these, DSAP is the most common. All forms of porokeratosis are characterized by the presence of a coronoid lamella, which is described as a raised rim of keratotic cells located at the border or edge of a skin lesion [2].

The appearance of linear porokeratosis is typically distributed along dermatomal or Blaschko lines. These lesions are generally unilateral in nature [3]. In addition to their observed effects and presentations, porokeratosis subtypes have demonstrated the potential to transform into malignancies, such as basal cell carcinoma and squamous cell carcinoma [4]. Porokeratosis presentations on average have a 7.5% chance of becoming malignant [5]. The most common resulting malignancies are squamous cell carcinomas; however, basal cell carcinomas and melanomas have also been observed [1,5].

The patient had previously demonstrated success with using topical cholesterol 2%/lovastatin 2% ointment to treat the linear porokeratosis lesions on his right arm, which served as our control [6]. To our knowledge, topical lovastatin 2% monotherapy has never been directly compared to the efficacy of topical cholesterol 2%/lovastatin 2% therapy in a split study in linear porokeratosis. Due to his unique case involving the bilateral distribution of porokeratosis, we compared the efficacy of his prior dual treatment to lovastatin 2% monotherapy.

## Case presentation

A 61-year-old male presented with asymptomatic thin, scaly, erythematous plaques in a linear distribution on the bilateral upper extremities. He reports these lesions initially presented at three years of age and were localized to the proximal aspect of his right upper extremity; however, they eventually progressed to his left upper extremity. There was no family history of similar conditions. A biopsy revealed a coronoid lamellae. Diep et al. published the histopathologic presentation confirming the diagnosis of linear porokeratosis in the same patient [6]. Following his diagnosis, he was prescribed topical clobetasol ointment but was unable to tolerate this treatment due to a painful burning sensation upon application. Calcipotriene cream and diclofenac gel were applied for six months, but no improvement was noted. Subsequently, the patient was started on a trial of cholesterol 2%/lovastatin 2% ointment on his right arm twice a day. After 12 weeks of treatment with this regimen, he noted improvement in the erythema and scaling of the lesions [6].

**Figure 1:**
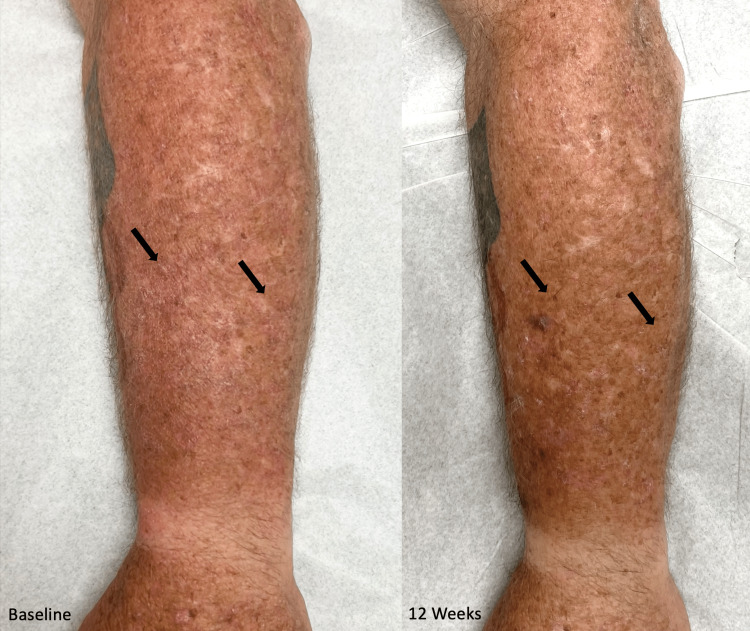
Linear porokeratosis before and after treatment with topical lovastatin monotherapy Visible improvement of the erythema and scaling of porokeratosis plaques (black arrows) on the left forearm after 12 weeks of treatment with lovastatin ointment monotherapy compared to baseline.

To differentiate the efficacy of topical cholesterol/lovastatin and topical lovastatin monotherapy, the patient received a 12-week course of topical lovastatin 2% ointment to apply to the left arm twice a day. After completing the trial, the hyperkeratosis was improved with a reduction in erythema and scale, but he still experienced post-inflammatory hyperpigmentation that was expected to resolve with time. The improvement in appearance was comparable to the results of the cholesterol 2%/lovastatin 2% ointment previously applied to his right arm. Throughout the duration of the trial, the patient similarly noticed a significant reduction of erythema and scaling of his lesions, as well as improved skin texture and appearance (Figure 1). No adverse effects were reported with the lovastatin monotherapy.

## Discussion

While the exact mechanism is currently unknown, porokeratosis has been observed to be caused by or correlated with exposure to UV light, immunosuppression, medication side effects, and autoimmune disorders, such as HIV and Crohn’s disease [7]. There have also been observations of possible genetic factors and predispositions that can lead to porokeratosis; these appear in a pattern of autosomal dominant inheritance patterns with observed mutation loci on chromosomes 1, 12, 15, 16, and 18 [7].

Common treatments for linear porokeratosis are similar to those used to treat other clonal keratinocyte disorders. However, porokeratosis does not currently have a standard of care since most reported data are derived from individual case reports and results have been variable. Many therapies have been attempted, and some have shown promising outcomes in attaining the resolution of lesions. In 2017, Weidner et al. performed a systematic review of existing case reports and case series describing various treatment modalities and their outcomes in treating variants of porokeratosis [8]. They concluded from their review that linear porokeratosis was observed to have the best therapeutic effects from topical or systemic retinoids. However, systemic retinoids can be costly and have known side effects affecting a patient’s quality of life that should be considered prior to starting treatment.

Grover et al. compared the efficacy of topical tretinoin and 5-fluorouracil (5-FU) in a 19-year-old male with linear porokeratosis. He had extensive involvement in his left upper and lower extremities [9]. The patient previously used several unknown topical therapies without improvement. A three-month split study of 5-FU 5% cream was applied twice daily to the left arm, while tretinoin 0.5% gel was applied at night to the left leg. The patient initially complained of mild discomfort due to the worsening of lesions, as well as marked dryness and scaling in the tretinoin-treated areas. Similarly, the patient developed extensive itching, hyperpigmentation, and areas of superficial ulceration in the 5-FU treated areas; this caused temporary discontinuation of treatment. The intolerability of 5-FU side effects is frequently observed to cause medication non-compliance [10].

In addition to fluorouracil and vitamin A derivatives, treatments such as vitamin D analogs and topical corticosteroids are often tested, aimed at reducing inflammation or the scaly appearance of the skin [5]. Other treatments are mainly focused on promoting lesional destruction through the use of diamond fraise dermabrasion, carbon dioxide lasers, and photodynamic therapy (PDT) [11]. 

Nguyen et al. described a case of a 51-year-old male with porokeratosis of the forehead, dorsal left hand, and upper right arm. The patient underwent ablative fractional carbon dioxide laser surfacing with three passes of the laser at 70 mJ with 35% coverage and one final pass at 50 mJ with 30% coverage along the edges. A formulation of 15% L-ascorbic acid, 1% alpha-tocopherol, and 0.5% ferulic acid was applied immediately after, and the patient was advised to reapply this solution twice daily for two days [12]. After two months, the lesions resolved without signs of recurrence. While this treatment is effective, it can be painful and costly for patients due to a lack of insurance coverage. 

Garcia-Navarro et al. described a case of a 13-year-old male with linear porokeratosis of the posterior right lower leg. There was no improvement after the application of calcipotriol ointment over a one-year period [13]. Showing no signs of improvement, the patient later underwent photodynamic therapy (PDT) treatment. First, the patient underwent the removal of any superficial scales with a surgical blade, and then methyl aminolevulinate hydrochloride cream 160 mg/g was applied to the area. The leg was subsequently subjected to red light for nine minutes. A second treatment was performed one month later. Burning sensations were reported with PDT treatment. After 11 months, the area showed no signs of recurrence. It is worthwhile to mention that PDT has been reported to have variable outcomes in the resolution of porokeratosis lesions [8]. Residual hyperpigmentation is a commonly reported side effect. Additionally, PDT is unlikely to be covered by insurance and may present as a costly option for treatment.

Recently, there have been promising cases highlighting a potential new therapy utilizing compounded topical cholesterol 2%/lovastatin 2% [14]. A literature search using the keyword “topical cholesterol lovastatin” on PubMed yielded eight articles citing successful treatments of DSAP or linear porokeratosis. Atzmony et al. tested topical cholesterol 2%/lovastatin 2% over three months on five porokeratosis patients, one with DSAP, two with porokeratosis palmaris et plantaris disseminata, and two with linear porokeratosis. It was noted that the compounded topical cholesterol lovastatin provided better results than the cholesterol alone for all porokeratosis subtypes [15].

Trials of cholesterol 2%/lovastatin 2% combination therapy have been more routinely investigated, due to the hypothesis that both pathways are involved in the pathogenesis of linear porokeratosis. There are two proposed theories for the cutaneous manifestation of porokeratosis lesions. One theory proposes a loss of function mutation in the phosphomevalonate kinase (PVMK) and mevalonate diphosphate decarboxylase (MVD) genes in the cholesterol synthesis pathway, leading to cholesterol depletion [15]. It is believed that this depletion affects the stratum corneum by causing premature apoptosis of keratinocytes and inducing porokeratosis formation. Another proposed theory discusses the accumulation of mevalonate toxic metabolites. In the functional cholesterol synthesis pathway, PMVK and MVD convert mevalonate into cholesterol. However, PMVK and MVD mutations disrupt this pathway and cause an upstream accumulation of mevalonate, which is thought to stimulate the innate immune response and cytokine production [16]. These factors may also induce porokeratosis lesions.

Ugwu et al. demonstrated the successful use of topical lovastatin 2% monotherapy in a patient diagnosed with DSAP of the upper and lower extremities, a condition closely related to linear porokeratosis [17]. In this study, the patient was started on a four-week regimen utilizing lovastatin 2% ointment, which resulted in the full resolution of his skin lesions. This therapy was implemented based on the theory that mevalonate metabolites had a more prominent role in the formation of porokeratosis.

The theory of mevalonate metabolites as the primary cause of porokeratosis formation is further supported by a randomized clinical trial published by Santa Lucia et al. in March 2023. They described the outcomes of their study comparing topical cholesterol 2%/lovastatin 2% and topical lovastatin 2% monotherapy on 31 DSAP patients over a 12-week period. They analyzed 17 participants using the lovastatin-cholesterol cream and 14 participants using the lovastatin cream. They determined the addition of cholesterol yielded little to no benefit as both groups saw similar results [18]. Although there are currently no randomized controlled trials on the applications of lovastatin monotherapy for linear porokeratosis, the similarity in pathogenesis between subtypes can be hypothesized to work with similar efficacy as observed in DSAP patients.

## Conclusions

Our study aimed to evaluate the use of lovastatin 2% monotherapy in linear porokeratosis, as there are currently no existing cases reported in the literature. At the conclusion of the 12-week trial, the patient demonstrated equivocal improvement with lovastatin 2% monotherapy compared to cholesterol 2%/lovastatin 2% dual therapy. This finding supports mevalonate being a source of the pathogenesis of porokeratosis. Based on this outcome, lovastatin 2% monotherapy may be considered when treating other variants of porokeratosis. In addition to tolerability, which enhances therapeutic compliance, compounded lovastatin ointment presents a reasonable option at an affordable price point.
